# Effects of ecological factors on the antioxidant potential and total phenol content of *Scrophularia striata* Boiss

**DOI:** 10.1038/s41598-019-52605-8

**Published:** 2019-11-05

**Authors:** Zahra Zargoosh, Mansureh Ghavam, Gianluigi Bacchetta, Ali Tavili

**Affiliations:** 1 Department of Range and Watershed Management, Faculty of Natural Resources and Earth Sciences, University of Kashan, Kashan, Iran; 2 Hortus Botanicus Karalitanus (HBK), University of Cagliari, Cagliari, Italy; 3 Department of Reclamation of Arid and Mountainous Regions, Faculty of Natural Resources, University of Tehran, Karaj, Iran

**Keywords:** Biochemistry, Chemical modification

## Abstract

*Scrophularia striata*, commonly known as figwort, is one of the most important medicinal plants that mainly grows in cold regions of the Zagros Mountains (West of Iran). Although the chemical composition of this plant species has not yet been explored, people living in Ilam province (W Iran) have used it for many years to treat different illnesses. The present study aims to analyze the effect of some ecological factors on the antioxidant potential and the amount of phenol present in this plant species, using a random factorial design with two factors (elevation and region) and three replicates. The fruits of the plant were gathered from three different elevations. They were collected from three regions of the Ilam province (Badreh, Dareshahr, and Dehloran) in June 2016, when the fruits appear. Moreover, to analyze different soil chemical and physical features, soil samples were gathered from a depth of 0.5 m under the shrubs. The antioxidant action of the methanol extract from the plant samples and the total amount of phenol compounds were measured using 2,2-Diphenyl-1-picrylhydrazyl (DPPH) and the Folin-Ciocalteu method, respectively. The results showed that the effects of site and elevation, and the interaction between these factors, on the antioxidant potential and total phenol amount were significant with a probability of error of 1%. The maximum extract efficiency (19.37 ± 3.07%), antioxidant potential (126.5656 ± 0.96 µg/mL), and total amount of phenol (55.7689 ± 3.17 µg/mL) were obtained from Dareshahr at an elevation of 600 m above mean sea level. The minimum amount of total phenol (24.6544 ± 3.21 µg/ml) was recorded at the lowest elevation of Badreh, at which phosphorus, potassium, organic carbon, organic material, nitrogen, acidity, lime, and silt were present at the lowest amount. However, the antioxidant activity and total amount of phenol had a strong direct correlation in the two districts of Dareshahr and Badreh, but were reversely and strongly correlated in Dehloran. Therefore, it can be stated that *Scrophularia striata* has the potential for antioxidant activity, however, the complexity of the effect of ecological factors on one hand, and the emergence of different chemical processes in the plant under such effects on the other hand, has led to the synthesis of different compounds with antioxidant potential in the plant in different regions.

## Introduction

For many years, natural remedies, especially medicinal plants, were the basis and, in some cases, the only treatment for illnesses, and their raw materials were used in the pharmaceutical industry^1^.

According to World Health Organization statistics, approximately 80% of the world’s population prefers primary health care to use herbal extracts or their active ingredient^2^. Plants and their compounds have the potential to replace chemical drugs and have fewer side effects than chemical drugs^3^.

Antioxidants, on the one hand, reduce the risk of cardiovascular and stroke patients, and on the other hand, prevent the progression of cancer caused by damage to the DNA^4^. Despite the presence of various antioxidants in the plasma, the immune system alone cannot eliminate the free radicals produced in the body, hence the need to provide antioxidants from external sources that are supplied through food sources^5^. Therefore, strong antioxidants with lower toxicity and a higher efficacy are an inevitable necessity.

Because that plant species are one of the important sources of antioxidants, research is on the rise. Plants that are rich in antioxidant compounds can protect cells from oxidative damage^6^. Natural antioxidants increase the strength of the antioxidants in the plasma and decrease the incidence of some diseases, such as cancer, heart disease, and stroke^7^.

Antioxidants are biologically active compounds that protect the body from damage caused by active oxygen species, active nitrogen, and active chlorine, which cause disease^8^.

The plant’s antioxidant compounds are mainly phenolic and include compounds such as tocopherols, carotenoids, phenolic acids (benzoic acid derivatives and cinnamon acids), flavonoids, and dipropenes^9^. Secondary plant-derived metabolites, including phenolic compounds, have a potent potential to clear free radicals that exist in all parts of the plant, such as the leaves, fruits, seeds, roots, and skin^10^.

Although the production of active ingredients in medicinal plants is guided by genetic processes, it is also strongly influenced by environmental factors. Therefore, environmental factors cause changes in the growth of medicinal plants, as well as the quantity and quality of their active ingredients, such as alkaloids, glycosides, steroids, and essential oils^11^.

In natural ecosystems, the determinants of production, other than species, are climate, soil, and geographic location. Each of these factors can have a major impact on increasing or decreasing the quantity and quality of plant performance^12^.

Researchers believe that many factors, such as water, air, soil, elevation (asl), and differences between species, extraction methods, and antioxidant measurements affect the amount of secondary metabolites in plants, including phenol and flavonoids. Overall, the antioxidant properties and the effects of habitat on the amount of secondary metabolites have been emphasized^13^.

The use of wild plant species in natural habitats, which, in addition to being ecologically compatible, can effectively synthesize secondary and active substances in environmental stress conditions, in the prevention and treatment of diseases in recent years has gained a special place in medical science^14^.

*Scrophularia striata* Boiss., which grows in Ilam province and parts of Khuzestan province^15^, is a perennial plant, 30–90 cm in height, with numerous stalks, small purplish flowers, and a flowering time between June and July^16^.

For many years, this plant has been experimentally used in various forms such as boiled, fumigated, edible, and poultice in the treatment of various diseases such as inflammation, eye and ear infections, skin burns, infectious ulcers, pain, and gastrointestinal disorders. Also, in other parts of Iran (Sistan and Baluchestan, Bushehr and Abadan) it has traditionally been used to treat allergies, rheumatism, and chronic inflammation^17–20^.

The present study was designed and implemented due to the importance of *Scrophularia striata* in traditional medicine among people in Ilam province. The aims of this study were (1) to assess the effect of some ecological characteristics on the antioxidant potential and phenolic compounds in *Scrophularia striata*, and (2) to identify the best habitat to achieve the highest antioxidant properties among the different habitat conditions in Iran.

## Materials and Methods

### Selected sites

*Scrophularia striata* is mainly distributed in the province of Ilam. This province is located in the western part of Iran, with a region of 20,150 km^2^. It is one of the semi-humid mountainous areas of Iran^21^. To select the sampling sites, at first, habitats of the plant were identified through field surveys. Then, considering the different environmental factors, three regions, namely Dareshahr, Badreh, and Dehloran, with a distance 35 km from together, were selected. The geographic and climatic characteristics of the study sites are shown in Tables 1 and 2. In each site, according to the presence and abundance of species in the same direction (northeast), three elevations at 100 m intervals were selected. Hence, the elevations in the Dareshahr site were 500, 600, and 700 m, in the Dehloran site 164.5, 264, and 364.5 m, and in the Badreh site 732, 832, and 932 m.

**Table 1:** Geographic characteristics of sampling sites.

Site	Longitude	Latitude	Highest altitude (m)	Lowest altitude (m)
Badreh	N 47° 11.58′ 74″	E 33° 11.29′ 70″	934	732
Dehloran	N 47° 17.42′ 08″	E 33° 43.17′ 34″	364	164
Dareshahr	N 47° 23.2′ 37″	E 33° 6.19′ 28″	700	500

**Table 2:** Climatic parameters of the sampling sites.

Site	Average rainfall (mm)	Average temperature (°C)	Maximum temperature (°C)	Minimum temperature (°C)	Relative mean ghost (%)	Maximum relative humidity (%)	Minimum relative humidity (%)
Badreh	385.5	20.8	27.7	13.9	40	54	26
Dehloran	297.8	26.2	32.3	20.2	38	51	26
Dareshahr	426.3	21.4	29.2	13.3	45	62	29

### Field sampling

#### Plant material

To sample the studied plant, in June 2016, coinciding with the appearance of fruit (based on the traditional use of these organs by people from the region and the presence of phenolic compounds in the plants at this time), nine populations were selected randomly from each site. 27 samples from each population were collected. In total, 9 × 27 = 243 samples at each site. The specimens were transferred to the laboratory after being harvested and exposed to open air to dry. Also, one sample of the whole plant was collected and pressed. The plant was identified by a botanist and recorded in the University of Kashan herbarium with code number 1210.

#### Soil samples

At each point of plant harvest, soil samples from the rootstock of the plant were collected at a depth of 0.5 m to study some of the physical and chemical properties of the soil and their relationship with the antioxidant properties of the plant. The specimens were first passed through a 2 mm sieve and then prepared for different experiments.

### Laboratory operation

#### Production of extract

Extracts of various samples were produced using a soxhlet device (Model 500 ml, Made in Iran) and a methanol solvent. For this purpose, 10 g of dried fruit was poured into a basket and placed in its own Timbal. Then, 350 mL of methanol solution was deposited in a 500 mL balloon. After the components were connected and the cold water was drawn and inlet, the extraction was started and lasted for 8 h. Then, a rotary evaporator (Heidolph Laborota 4003 Control Rotary Evaporator with Water Bath) was used to condense the extracted solution, which was subsequently transferred to an oven at 45 °C for 24 h. then to a vacuum oven (35 °C and pressure 20 mL of mercury) to dry the specimens. After 4 days, the dried extracts were separated from the plates using a spatula.

The extract yield was calculated based on the dry extract weight per 100 g dry matter. To prevent the degradation of the active ingredients, the extracts were transferred to impervious dishes and kept at 4 °C until further tests were performed.

#### Evaluation of antioxidant properties of the extract (DPPH test)

In this study, the ability of plant extracts to act as an antioxidant for the radical 2,2-Diphenyl-1-picrylhydrazyl (DPPH) was measured. This radical absorbs light at 517 nm and its intensity follows the Beyer-Lambert law. The reduction in absorption by this compound has a linear relationship with the amount of antioxidant. Therefore, if the antioxidant agent is increased, DPPH is more consumed and its color changes from violet to yellow. To investigate the antioxidant property, it seems necessary to use a standard test. In this study, butylated hydroxy toluene (BHT) was used as the standard control. To ensure the accuracy of the test and to reduce the error rate, each of the tests of standard extract and the control was repeated three times, and after averaging the data, the IC50 of each of the samples was determined.

The following solutions were prepared for this test: The solution of DPPH: 7.4 mg of solid DPPH was weighed, poured into a 50 mL volumetric flask, and then dissolved in 50 ml of methanol. Since the solution of DPPH degrades and decomposes in light, a dark volumetric flask was used at the end of the work. The color of the resulting solution over time changes from purple to yellow due to its rapid recovery in the environment. DPPH was then stored in a refrigerator at 4 °C. Also, due to DPPH’s corrosivity, it was essential to observe safety precautions when using it. Typically, after preparing the solution of DPPH, a preliminary test was performed to ensure its high standard. One milliliter of DPPH solution was made and 1 mL of methanol was poured into a 5 mL dark balloon and stored at 25 °C for 30 min. After approximately 30 min, using a spectrophotometer (Model UV-2100, Made in the USA), the absorption of the solution was measured at 517 nm. If the absorption read between 1.1 and 1.7, the solution of DPPH was used in the test.

The solution of standard of Butylated hydroxytoluene (BHT): In order to compare the results obtained for the plant samples with the standard BHT specimen, to determine the accuracy of the process, and to assess the antioxidant activity of the standard BHT, 25 mg of standard of BHT was weighed and completely dissolved in 25 ml of methanol in a 25 mL volumetric flask. Then, the solution of BHT was made with a concentration of 1 mg/mL. In the next step, concentrations of 0.8, 0.5, 0.25, 0.1, 5 × 10^−2^, 5 × 10^−3^, and 5 × 10^−4^ mg/mL of the solution of from the stock solution were prepared by dilution with methanol in 8 volumetric flasks of 10 mL.

Solutions of extract: To prepare the base solution, 25 mg of each of the methanolic extracts from the plant samples was weighed, poured into a 25 mL volumetric flasks, and mixed with methanol. All components of the extract were completely dissolved in methanol and the resulting solution was homogeneous. The stock solution was prepared at a concentration of 1 mg/mL for each of the samples. Then, solutions with concentrations of 0.8, 0.5, 0.25, 0.1, 5 × 10^−2^, 5 × 10^−3^, and 5 × 10^−4^ mg/mL were prepared. One milliliter of each of the above solutions was poured into the corresponding dark volumetric flask. Then, 1 mL of the DPPH solution was added to each of the dark volumetric flasks and mixed. The dark volumetric flasks were stored at 25 °C for 30 min.

The solution of control: 1 mL of methanol and 1 mL of DPPH solution were added to a 5 mL balloon and mixed.

Finally, after 30 min, the absorption of the solutions at a 517 nm wavelength was read using a UV/vis spectrophotometer, starting with the control solution and then reading the samples in order of increasing concentration. At the end of the calculation, the percentage of antioxidant contained was calculated according to the plot of the negative logarithm of the concentration in EXCEL, and the IC50 was calculated in micrograms per milliliter. The percentage of inhibition was calculated using the following equation.

#### Measurement of the total amount of phenolic compounds

In this study, the Folin-Ciocalteu method was used to measure the total amount of phenolic compounds. The following solutions were prepared for the Folin–Ciocalteu test:

1. Gallic acid standard solutions: 1.1, 2.2, 3.3, 4.4, 5.5, 6.6, 7.7, 8.8, 9.9, 10.10, and 11 mg of gallic acid were weighed and placed in 11 separate test tubes. One milliliter of ethanol was added to each tube containing gallic acid. Three milliliters of distilled water was added to each of 11 5 mL volumetric flasks, then 0.01 mL (10 μL) of each gallic acid solution was added to one of the 11 volumetric flasks, separately. In the next step, 0.1 mL (100 μL) of Folin-Ciocalteu Merck was added to the volumetric flasks and after 3 min, 0.3 mL (300 μL) of sodium carbonate solution 2% was added, and then the volumetric flasks with ethanol were reached to the volume and mixed. The volumetric flasks were placed at 25 °C for 2 h.
2. Control sample for the gallic acid samples: 100 mL ethanol instead of the gallic acid solution was used and the same steps as above were repeated.
  After 2 h, the wavelength of the spectrophotometer device was adjusted on 760 nm. At first, the device was zeroed with the control solution, then the absorbance of each of the solutions was read three times and the average of the three readings taken. From the absorption assays, the absorbance graph of the concentration (μg) was plotted in the EXCEL and the line equation was calculated (Fig. 1(.

**Figure 1:**
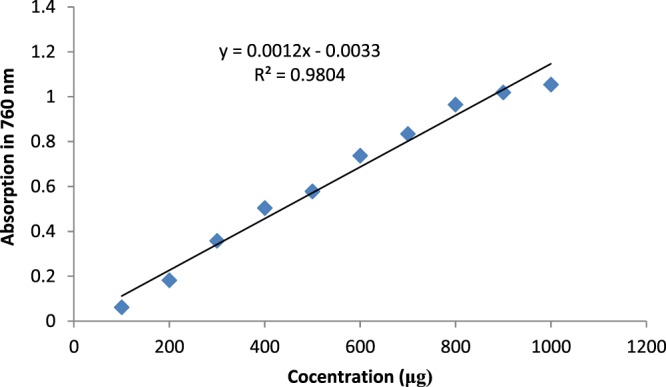
Gallic acid standard curve.
3. Extract solutions: After the total phenol content of the gallic acid standards was analyzed, a total phenol test was performed for the plant samples. For this purpose, 10 mg from each sample of the herbal extract was weighed, placed in a test tube, and 2 mL of DMSO solution was added to completely dissolve the sample. For each extracted sample, three 5 mL the volumetric flasks were used for three replicates. Each of the volumetric flasks contained approximately 3 mL of distilled water and 0.20 mL (20 μL) of each extracted sample. One hundred microlitres of Folin–Ciocalteu reagent was added to the volumetric flasks and after 3 min, 0.3 mL (300 μL) sodium carbonate 2% was added and then distilled water was added and the volumetric flasks were reached to the volume.
4. Control solution: In addition to the sample volumetric flask, a 5 mL control volumetric flask was used. Instead of 0.20 mL of the extract, 0.20 mL of dimethyl sulfoxide (DMSO) solution was added. The solutions made in the volumetric flasks were uniformly homogeneous and placed at 25 °C for 2 h. After 2 h, the absorbance of each solution was read three times using a spectrophotometer apparatus at a wavelength of 760 nm. For each plant sample, the average of the three readings was calculated and used in the equation of the standard gauge line obtained from the equation of the gallic acid line, and the concentration of the phenolic compounds in the extract of the plant was calculated using the amount of gallic acid, measured in micrograms:

$$\begin{array}{rcl}{\rm{Absorbance}} & = & 0.0012\times {\rm{galic}}\,{\rm{acid}}\,({\rm{\mu }}g)+0.0033\\ {R}^{2} & = & 0.98\end{array}$$

#### Soil

To determine the soil physical and chemical properties, the following methods were used: texture was determined using a hydrometric method, soil acidity using a pH meter, electrical conductivity using an EC meter, carbon, and organic matter were determined according to the methods described by Walkley & Black, the nitrogen content was determined using a Kjeldahl method, limestone using an acid and alkali titration method, soil plaster content using acetone, phosphorus using Olsoun, and potassium using the ammonium acetate extraction method.

#### Statistical analysis

The present study was carried out in a factorial arrangement in a completely randomized design with two factors (elevation and region) and three replications. The statistical analysis was performed using SPSS software. First, the normality of the statistical variables was investigated using a Kolmogorov-Smirnov test, and after ensuring the normality of the data, the variance of the data (soil and plant quantities) was analyzed using an F-test and a comparison of the means using a Duncan test with a probability level was 1% error was performed. A Pearson correlation test was used to study the correlation between plant attributes, elevation, and soil properties. The sample size was 243. Also, multiple regression was used to determine the relationship between soil characteristics and extracts.

## Results

### Effect of elevation and site

#### Extract efficiency

Based on the results of the analysis of variance, the yield of the extract of *S. striata* from the studied sites and elevations was significantly different. The effect of the site and the interaction of the site and elevation on extract yield was significant (P ≤ 0.01). In addition, the analysis of variance of antioxidant capacity in terms of IC50 (ability to neutralise 50% of DPPH free radicals) and the total amount of phenolic compounds in *S. striata* showed that the effect of site, elevation, and the interaction of site and elevation was significant at a probability level of 99% (Table 3).

**Table 3:** Analysis of variance of site and elevation effects on extract yield, antioxidant capacity, and total phenol levels of *S. striata*.

Source of variation	df	MS		
		Yield of Extract	Antioxidant capacity	Total phenolic compounds
Site	2	40.528**	22634.582**	527.663**
Elevation	2	5.898^ns^	61571.571**	41.536**
Site × Elevation	4	15.828**	24963.991**	286.258**
Error	18	2.982	2.933	5.083

ns: Not significant, **: 1% level of probability is significant.

**Figure 2:**
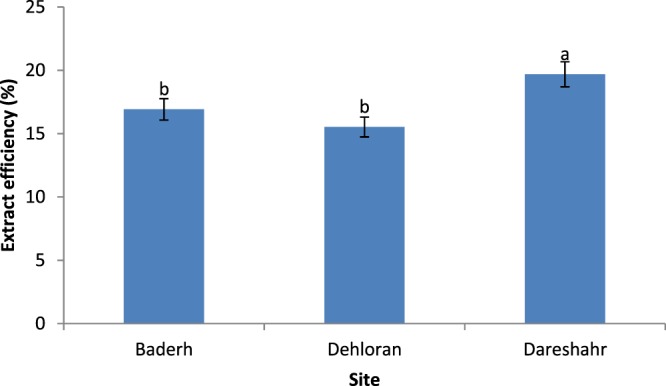
Comparison of the mean effect of site on the yield of extracts of *S. striata*

The results of the comparison of the average yield of extracts from different sites showed that the highest extract yield came from the samples from Dareshahr, whereas there was no significant difference between the two other sites for the yield extract (Fig. 2).

In addition, the results shown in Table 4 indicate that the third elevation at the first site (Badreh) with 19.04 ± 1.01%, and all elevation classes at the third site (Dareshahr) had the highest yield, and the first elevation of the first site (Badreh) and the third elevation of the second site (Dehloran) had the lowest yield.

**Table 4:** Comparison of the mean of the interaction of site × elevation on the yield of extracts of *S. striata*.

Site	Elevation (m a s.l.)	Mean (%) ± SD
Badreh	732	**13.73** ± **0.31**^**c**^
	832	**18.00** ± **1.11**^**ab**^
	932	**19.04** ± **1.01**^**a**^
Dehloran	164.5	**15.60** ± **0.60**^**bc**^
	264	**17.43** ± **0.68**^**ab**^
	364.5	**13.53** ± **0. 23**^**c**^
Dareshahr	500	**20.71** ± **3.50**^**a**^
	600	**19.37** ± **3.07**^**a**^
	700	**19.00** ± **1.40**^**a**^

The different letters in each column indicate a significant difference based on Duncan’s multiple range test at the 5% level.

#### Antioxidant capacity measured using DPPH

The results of the analysis of variance of antioxidant capacity in terms of IC50 (ability to neutralize 50% of DPPH free radicals) of *S. striata* showed that the effect of site, elevation, and the interaction of site and elevation were significant (P ≤ 0.01) (Table 5).

**Table 5:** Comparison of the mean effect of the interaction of site × elevation on the antioxidant capacity of *S. striata* according to the IC50 using the DPPH test.

Site	Elevation (m.)	Mean (%) ± SD
Badreh	732	**400.1544** ± **3.37**^**a**^
	832	**399.4911** ± **1.53**^**a**^
	932	**200.5056** ± **1.89**^**d**^
Dehloran	164.5	**400.8411** ± **3.26**^**a**^
	264	**200.6600** ± **1.59**^**d**^
	364.5	**195.9967** ± **2.35**^**e**^
Dareshahr	500	**317.4444** ± **1.63**^**b**^
	600	**126.5656** ± **0.96**^**f**^
	700	**262.2033** ± **1.20**^**b**^

The different letters in each column indicate a significant difference based on Duncan’s multiple range test at the 5% level.

**Figure 3:**
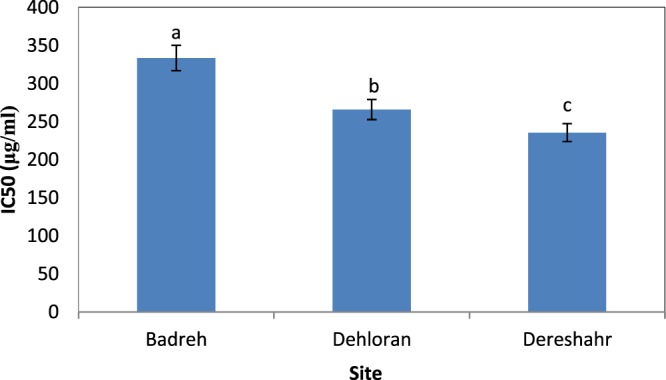
Comparison of the mean effect of site on the antioxidant capacity of *S. striata* according to the IC50 using the DPPH test.

**Figure 4:**
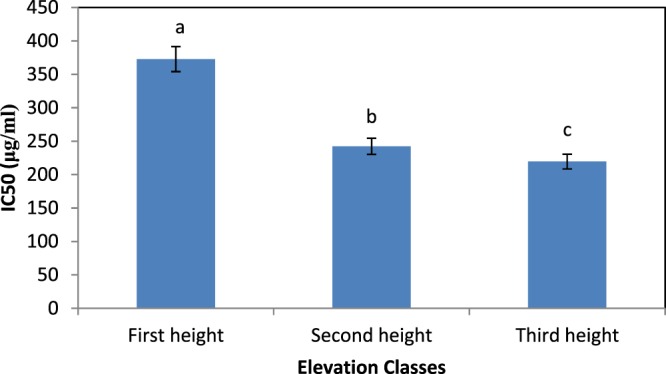
Comparison of the mean effect of elevation on the antioxidant capacity of *S. striata* according to the IC50 using the DPPH test.

Figure 3 shows that the highest IC50 belonged to Baderh (333.33 μg/mL) and the lowest to Dareshahr (233.48 μg/mL). However, at the first altitude, the highest IC50, and the highest elevation, the lowest IC50, were recorded (Fig. 4).

In addition, according to the results presented in Table 5, the first and second elevation of Baderh produced 400.1544 ± 3.37and 399.4911 ± 1.53 μg/mL, respectively, and the first elevation of Dehloran produced 400.8411 ± 3.26 μg/mL and had the highest IC50, and the second elevation of Dareshahr with 126.5656 ± 0.96 μg/mL had the lowest IC50.

#### Total phenol content determined using the Folin-Ciocalteu method

**Figure 5:**
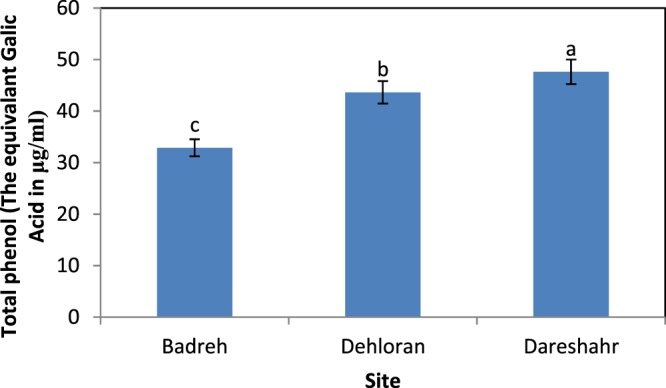
Comparison of the mean effect of site on the total phenol content of *S. striata*.

**Figure 6:**
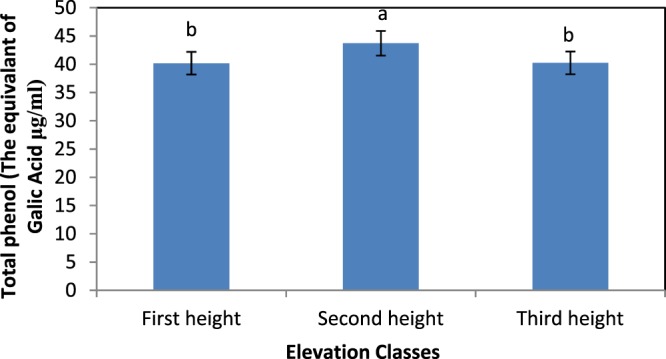
Comparison of the mean effect of elevation on the total phenol content of *S. striata*.

The analysis of variance of the total phenolic compound content of *S. striata* showed the effect of site, elevation, and the interaction of site and elevation at the level of probability of 1% error (Table 3). The comparison of the averages from the different sites (Fig. 5) indicated that the Dareshahr site, with a mean of 47.62 µg of gallic acid in 1 g of dry matter, and Badreh with 32.88 µg of gallic acid in 1 dry matter, had the highest and lowest phenol content, respectively. Furthermore, according to Fig. 6, the middle altitudes with a mean of 43.71 µg of gallic acid in the 1 g of dry matter of extract had the highest phenol content.

The results shown in Table 6 show that the highest total phenol content (55.7689 ± 3.17 μg of gallic acid in 1 g of dry matter of extract) belonged to the second elevation in Dareshahr, and the lowest (24.6544 ± 3.21 μg of gallic acid in 1 g of dry matter of extract) belonged to the first elevation at the Badreh site.

**Table 6:** Comparison of the mean effect of the interaction of site × elevation on the total phenol content of *S. striata*.

Site	Elevation (m)	Mean (%) ± SD
Badreh	732	**24.6544** ± **3.21**^**g**^
	832	**29.8889** ± **1.58**^**f**^
	932	**44.1011** ± **3.65**^**c**^
Dehloran	164.5	**48.8244** ± **2.93**^**b**^
	264	**45.4878** ± **1.2**^**c**^
	364.5	**36.6011** ± **2.27**^**e**^
Dareshahr	500	**47.0656** ± **5.05**^**bc**^
	600	**55.7689** ± **3.17**^**a**^
	700	**40.0289** ± **12.67**^**d**^

The different letters in each column indicate a significant difference based on the Duncan multiple range test at the 5% level.

### Impact of soil and elevation characteristics

The correlation results of different soil characteristics and the elevation above the sea level with different quantities of plant extract are listed separately for the studied sites in Table 7.

**Table 7:** Correlations of elevation and soil characteristics with extract efficiency, antioxidant capacity, and total phenol content in the studied sites.

Site	Correlation	Silt	Clay	Sand	Caso_4_	Lime	Nitrogen	Organic matter	Organic carbon	Potassium	Phosphorus	Electrical conductivity	Acidity	Elevation
Badreh	Extract efficiency	0.936**	−0.708*	−0.939**		0.924**	0.944**	0.957**	0.938**	0.553^ns^	0.940**	−0.921**	0.223^ns^	0.884**
	Antioxidant capacity	−0.502^ns^	0.106^ns^	0.546 ^ns^		−0.482^ns^	−0.737*	−0.675*	−0.704*	−0.984**	−0.752*	0.448^ns^	0.886**	−0.889**
	Total phenol	0.679^*^	−0.231^ns^	−0.724*		0.667*	0.873**	0.815**	0.835**	0.890**	0.853**	−0.663^ns^	0.725*	0.946**
Dehloran	Extract efficiency				0.901**	0.856**	−0.969**	−0.969**	−0.969**	−0.765*	−0.345^ns^	0.259^ns^	0.044^ns^	**−0.513** ^**ns**^
	Antioxidant capacity				−0.308^ns^	0.507^ns^	−0.200^ns^	0.201^ns^	−0.200 ns	−0.572^ns^	−0.710*	0.975**	−0.999**	−0.874**
	Total phenol				0.463^ns^	0.914**	−0.793**	−0.793**	−0.793**	−0.914**	−0.598^ns^	0.801**	−0.655^ns^	−0.914**
Dareshahr	Extract efficiency	0.268 ^ns^	−0.179^ns^	−0.168^ns^		0.002^ns^	0.301^ns^	0.301^ns^	0.301^ns^	0.105^ns^	−0.219 ^ns^	0.116^ns^	0.064^ns^	**−0.290** ^**ns**^
	Antioxidant capacity	0.881^**^	0.768*	−0.944**		−0.858**	0.301^ns^	0.308^ns^	0.308^ns^	−0.370^ns^	0.198^ns^	0.966**	0.871*	**−0.281** ^**ns**^
	Total phenol	−0. 788^*^	−0.646 ns	0.832**		0.924**	0.408^ns^	0.402^ns^	0.402^ns^	0.828**	−0.622^ns^	−0.831**	0.930**	**−0.429** ^**ns**^

**Significant at the probability level of 1% error. *Significant at 5% probability level. ns: not significant.

Also, Table 8 shows the regression equation for each of the plant quantities in different sites.

**Table 8:** Regression equation of extract efficiency, antioxidant capacity, and total phenol content in studied sites.

Site	Herbal quantity	Equation	F	R^2^
Badreh	Extract efficiency	Extract efficiency = +1.553 +3.794OM	75.857^**^	**0.916**
	Antioxidant capacity	Antioxidant = −2344.659–1.318 K–893.258 N +372.013 pH	608.730^**^	**0.977**
	Total phenol	Total Phenol = −47.846 + 0.097Elevation	60.067^**^	**0.946**
Dehloran	Extract efficiency	Extract efficiency = +20.760–60.099 N	109.536^**^	**0.940**
	Antioxidant capacity	Antioxidant = +3937.722–483.432 pH	4154.781^**^	**0.988**
	Total phenol	Total Phenol = +60.023–0.061Elevation	35.758^**^	**0.914**
Dareshahr	Extract efficiency	—		
	Antioxidant capacity	Antioxidant = −5015.205 + 340.368EC + 576.337 pH	178.614^**^	**0.983**
	Total phenol	Total Phenol = −382.615 + 53.952 pH	44.928^**^	**0.930**

**Significant at the probability level of 1% error.

#### Extract efficiency

The yield of extract in Baderh had a significant correlation with all variables (except acidity and potassium level). An indirect correlation was only found with electrical conductivity (Table 7). However, the amount of organic matter had the highest direct correlation with a yield of extract. The regression results confirm this correlation (Table 8).

In Dehloran, elevation, acidity, electrical conductivity, and soil phosphorus content were not correlated with the yield of plant extracts, and other characteristics of soil (other than lime) had an indirect correlation with this characteristic. Also, the highest indirect correlation with organic carbon, organic matter, and nitrogen was recorded (Table 7), which confirms the results of regression in Table 8.

It should be noted that based on the results of the correlation of extraction efficiency with all variables in Dareshahr, and the lack of a significant correlation of soil characteristics and elevation with a yield of extract, no regression model was introduced.

#### Antioxidant capacity

The results indicate that the antioxidant capacity in the Badreh site had was indirectly correlated with elevation and phosphorus, potassium, organic carbon, organic matter, and nitrogen levels. The regression equation represents these relationships (Tables 7 and 8).

In Dehloran, acidity had the highest indirect correlation with antioxidant capacity, which is seen in the resulting regression equation. It is worth noting that the elevation and amount of soil phosphorus were negatively correlated and the electrical conductivity positively correlated with this characteristic (Tables 7 and 8).

Based on the results shown in Tables 7 and 8, at the Dareshahr site, the antioxidant capacity was indirectly correlated with the lime and sand percentage. Also, the clay percentage, silt content, acidity, and electrical conductivity had a direct correlation. The regression equation represents these relationships.

#### Total phenol content

The results shown in Tables 7 and 8 showed that in the Baderh site, the total phenol content was significantly directly correlated with elevation and all soil properties, except for electrical conductivity and clay percentage. The total phenol content was significantly indirectly correlated with sand percentage. Also, elevation showed the highest correlation with the total phenol content of the plant. The regression equation shows this clearly.

This vegetative characteristic in Dehloran, except for acidity and phosphorus and gypsum, had a significant correlation with other variables. The regression results showed that only elevation above sea level had a significant relationship with the total phenol content.

In Dareshahr, the total phenol content was significantly directly correlated with acidity, lime content, potassium content, and sand percentage. However, it had a significant indirect correlation with electrical conductivity and silt percentage. Among these, acidity was found to have the highest correlation with this characteristic in the regression equation.

### Correlation between antioxidant capacity in terms of IC50 and the total phenol content

The results shown in Table 9 indicate that there is a strong negative correlation between antioxidant capacity and the total phenolic compound content in Baderh and Dareshahr, whereas there is a strong positive correlation in Dehloran with a 99% confidence coefficient.

**Table 9:** Comparison of antioxidant and phenol correlations in the studied sites.

Site	Correlation	Antioxidant capacity	Total phenol
Badreh	Antioxidant capacity according to IC50	1	−0.913**
	Total phenol		**1**
Dehloran	Antioxidant capacity according to IC50	1	0.672**
	Total phenol		**1**
Dareshahr	Antioxidant capacity according to IC50	1	−0.639**
	Total phenol		**1**

**Significant at the probability level of 1% error.

## Discussion

### Extract efficiency

The effect of site on the extract yield was significant and the highest yield of extract belonged to samples from Dareshahr^22^. reported different yields of the extract of *Thymus danesensis* in different sites. The effect of habitat on the amount of secondary metabolites in different herbs has been studied previously. In most cases, the role of habitat has been emphasized as a factor affecting the quantity and accumulation of secondary metabolites^23–25^, which is in agreement with the results of this study. The location a plant grows can affect the process of producing effective substances due to temperature and humidity changes^26^. The mechanisms underlying environmental effects on the accumulation of secondary metabolites is not properly understood. However, the environment influences the type and number of chemical reactions through its effect on the process of metabolite production and factors associated with the production process (eg. enzymes). The relative rainfall and relative humidity of the Dareshahr were reported to be 426.3 mm and 45% per year, respectively, which is higher than at the other two sites. However, no correlation was found between the yield of the extract from this site and soil characteristics and elevation, and there were no statistically significant differences between the different elevations. Thus, it can be said that climatic conditions (moderate semi-arid climate) are more dominant. It is noteworthy that the dominance by climatic conditions is much more pronounced than in Dehloran, where the climatic differences between the two sites (Dereshahr and Dehloran) are quite clear (Table 2).

However, the effect of the interaction between elevation and site on the yield of the extract was significant, and at the highest elevation of Badreh, the yield of the extract was statistically equal to Dareshahr. It is worth noting that the elevation of the sampling point was more than 900 m above sea level and the highest elevation of all the sampling sites. The direct correlation between elevation and efficiency in this site is a reason for this, since at high altitudes, radiation, especially UV-B, is increased^27^. However, the results of the study by^28^ on *Marrubium vulgare* L. showed a negative correlation between altitude and yield. The plant species and other ecological factors, including geographic location, might be factors affecting this difference in results. Changes in temperature gradient due to altitude change are the most important factors in plant life, and factors such as temperature, relative humidity, wind speed, available water content, and received radiation change with increasing or decreasing in elevation. The plant is also affected by biomass changes, and changes in altitude and the location of the altitude can change many of the ecophysiological reactions^29^. Increasing the elevation above sea level increases ambient light, reduces plant height, and increases the number of branches. High light intensity in comparison to natural light causes a general growth of branches, increasing the number of branches and lateral branches, thickening the branches, coloring and brightening the leaves, increasing the chlorophyll content, reducing stomatal breathing, and increasing the photosynthetic potential, and in these conditions, the dry weight yield increases.

However, based on the results of the regression, the increase in organic matter in this site (Dareshahr) increased the yield of the extract. Similar results by^30^ using *Mentha longifolia* L. were obtained in Marand habitats. The increase in organic matter was observed with increasing altitude in this site, with the lowest extract yield at the elevation of 732 m (133.73 ± 0.31%) and the highest yield (4.63 ± 0.03%) reached at an elevation of 932 m. To date, many studies have been conducted to investigate the interaction between vegetation and soil^31,32^ and have substantiated the correlation between vegetation and soil in their studies^33^. Organic matter is known to be one of the fertility elements of the soil due to its effects on the physical, chemical, and biological properties of the soil. Other features of organic materials include water absorption and preservation, and the prevention of erosion and the pollution of groundwater. The presence of more organic matter in the soil with optimum moisture storage, better root growth, and a gradual release of nitrogen, increased nitrogen uptake. However, due to the high amount of organic matter in the soil, the availability of soil phosphorus is likely to increase. Organic matter in the soil causes nutrients in the soil with a low solubility to be absorbed by organic matter and become usable and absorbable for the plant^34^. Soil organic matter increases the phosphorus content by increasing the solubility of insoluble phosphorus. It indirectly prevents phosphate precipitation at pH 6 to 9, which is inaccessible to the plant. However, the loamy texture of the soil at this elevation increased the absorption of water by the roots of the plants and, with increasing photosynthesis and plant growth, the percentage yield increased, which is consistent with the results of^35^. In Dehloran, the extract yield was positively correlated with gypsum and limestone levels and had a direct correlation with potassium, organic carbon, organic matter, and nitrogen, so that with the increase in nitrogen with increasing elevation, the extract yield decreased and at the third elevation, the site had the lowest returns. It seems that the different climatic conditions of Dehloran (warm and dry climate, annual rainfall of 279.8 mm which is less than the other two sites, relative humidity of 38% which is less than the other two sites, and the higher degree of heat) had a great impact on the absorption and efficiency of nitrogen use. Environmental conditions can affect nitrogen use efficiency. Temperature is one of the ecological factors limiting the growth of plants and the synthesis of effective materials by medicinal plants^36^. found similar results when studying the effects of the application of nitrogen in semi-arid and temperate cold conditions on the herb thyme. However, based on the hypothesis proposed by^37^, access to nutrients limits the production of secondary metabolites by plants, especially as the accumulation of substances in plant tissues increases and this resource is directly allocated to biochemical pathways. When the environmental conditions are favorable and sufficient nutritional elements are available to the plant, the theory of GDB (Growth-differentiation balance) management mode between growth and the production of secondary metabolites) leads to vegetative production and tends to produce protein by assigning photosynthetic materials. However, in unfavorable and poor environments when nutrients such as GDB nitrogen are restricted to production and not assigned to secondary metabolites and can be stored to protect and eliminate environmental hazards in later stages.

### Antioxidant activity

The results showed that the IC50 values varied from 400.8411 ± 26.36 to 126.556 ± 0.696 μg/mL in the different elevations of the different sites. Since IC50 is inversely associated with the anti-radical activity of the compounds, the lower the IC50, the higher the antioxidant activity^30^. Based on the results of this study, the effect of the site on the antioxidant activity of the plant was significant, which is consistent with the findings of^14,22,38–48^. Dareshahr, with the lowest IC50, had the highest antioxidant activity. The antioxidant compounds contained in plant extracts have several functions and their activity and mechanism of action strongly depend on their composition and environmental conditions, since these conditions affect the synthesis of plant chemicals that have antioxidant properties^49^. The plant’s habitat, as a result of climate change, can affect the formation of secondary active substances in the plant. The relative humidity and low temperature of this site are one of the factors affecting the increase in antioxidant activity^50^. in their review of the anti-oxidative activity of *Ferula assafoetida* L. emphasized this. In addition to climatic factors, soil conditions can be affected. At this site, there was a direct correlation between acidity, electrical conductivity, and the silt percentage of the soil with the IC50, and a strong inverse correlation between the lime and soil percentage with the IC50. Therefore, increasing the concentration of soil salts, as well as the heavier soil texture, hurts antioxidant activity. Although many studies have indicated that salinity induced by metabolic processes increases the level of phenolic, flavonoid, and oxidative enzymes^51^, according to our results, increased electrical conductivity in this site (Dareshahr) did not affect the plant’s antioxidant capacity, which is exactly in agreement with the study by^50^ and contradicts the results of^42,44,52^. The lowest amount of antioxidant activity was found in samples from the Baderh site. Although this site was climatically similar to the Dareshahr site, it seems that soil conditions determine the anti-oxidant property of the collected samples in this site relative to the Dareshahr. Soil properties are the effects of other environmental factors over time, and there is a strong correlation and close relationship between vegetation and soil, such that a change in the state of each one will have a profound effect on other functions of the ecosystem. The acidity in the Bardeh site with an average pH of 3.8 at the three altitudes was generally less acidic than the other sites of study, resulting in more alkalinity in the soil environment. As indicated in the regression equation for this site, increasing acidity is directly and significantly related to increases in IC50 and a reduction in antioxidant activity, however^42^, when examining *Gundelia tournefortii* L., showed that soil conditions with loamy tissue, higher acidity, higher EC (Electrical Conductivity), and a lower percentage of lime were considered as factors influencing the increase of antioxidant activity.

The effect of elevation on the quantity of this herbal was significant; as the elevation increased, the IC50 decreased and the antioxidant property increased^13,38,45,53,54^. achieved similar results. There was a significant correlation between the altitude above sea level and IC50 in the Badreh site. Hence, with increasing elevation, antioxidant activity increased and reached its highest content at the third altitude of the site. Altitude as a peripheral environmental factor is highly correlated with some of the constituents of the plant^55^. Research in other plants such as *Heracleum* sp. and *Arnebia euchroma* L. has shown a direct relationship between elevation and the consequent effects of environmental stresses on the amount of active ingredients and, most importantly, the enhancement of free radical inhibitory potency and the antioxidant capacity of the extract of these plants^56,57^. Therefore, it can be concluded that at high altitudes, due to the reduced temperature and increased exposure to UV radiation of the organ of responsible for the synthesis of antioxidant compounds, antioxidant activity increases with increasing altitude. In the Dehloran site, the strong correlation between elevation and IC50 was significant, therefore at the highest elevation, the highest antioxidant activity was observed for this site. It should be noted that the effect was not significant in the Dareshahr site.

Also, the interaction between elevation and site on antioxidant activity was significant, which is consistent with the results of^38^. The collected samples from the first and second elevation at Badreh and the first elevation at the Dehloran site have the highest IC50 values, which indicates a higher concentration of the extract is required to inhibit 50% of free radicals and thus less activity of the antioxidants. In the Badreh site, the antioxidant activity changed from its lowest level at 732 m to its highest level at 932 m above sea level. Temperature variation due to changes in elevation is one of the most important factors influencing the habitat of a plant^38^. In this site (Badreh), changes in soil properties with changes in elevation were among the factors affecting the reduction in IC50 and the increased antioxidant activity. It should be noted that the soil acidity at the third elevation was the lowest (8.16 ± 0.005), and the elements of phosphorus, potassium, and nitrogen increased to their highest level at sea level elevation. However, the antioxidant activity in the Dehloran site (with hot and dry climate was the least rainfall and higher temperature than the other two sites) in addition to the climate, under the influence of elevation and some characteristics of the soil were. In this site, the IC50 had a direct correlation with the electrical conductivity and had an inverse relationship with soil elevation, acidity, and phosphorus. Therefore, along with a decrease in the electrical conductivity and an increase in phosphorus and acidity with elevation, antioxidant activity increased. The amount of phosphorus in this site was 1.26% on average, which was the highest of all the sites. It can be said that the high phosphorus content of the Dehloran site compared to the Baderh site (despite the similarity between the climatic conditions of Badreh and Dareshahr) led to a decrease in the antioxidant properties of extracts from Dehloran^58^ in a study of the antioxidant activity of *Bunium persicum* Boiss., found an effect of phosphorus and nitrogen on the increase in antioxidant activity.

### Total phenol content

The findings indicate that the effect of the site on the total phenol content of the plant was significant^14,22,38–40,42–46,48,50,53,59^ achieved similar results with other plants. However^60^, noted in their study that the effect of the site on the total phenol content was not significant. The highest content of total phenolic compounds was observed in Dareshahr. Phenolic compounds are found in a wide range of plants. The production of these compounds, although under the control of genetic factors, are significantly affected by the environment^39^. Studies have shown that environmental factors, such as rainfall and average temperatures, as well as the concentration of nutrients in the soil, can alter the level of polyphenolic compounds^61^. Climatic factors, such as high rainfall and high relative humidity, and lower temperatures in the site might be a reason for the higher total phenol content in the site^50^ obtained similar results for *Ferula assafoetida* and^62^ for *Berberis vulgaris*. Also, the phenol content plants in this site were correlated with acidity, limestone, sand content, and soil potassium, and there was a direct correlation with electrical conductivity and silt percentage, which is evident in the regression equation. Therefore, lighter soil texture, reduced salinity, and increased lime and potassium increased the amount of plant phenolic compounds. In this site, the highest amount of potassium, sand and lime percentage was recorded^50^ also noted an increase in phenol in *Ferula assafoetida* in line with a decrease in salinity. The lowest total phenol content was observed in Baderh, indicating the dominance of other ecological factors (elevation and soil) in decreasing the amount of phenol relative to the Dareshahr. Elevation; acidity; the concentration of phosphorus, potassium, organic carbon, organic matter, and nitrogen; the lime percentage, and silt percentage with a direct correlation, and the sand percentage with a reverse correlation, controlled the total phenol content. Among these characteristics, it seems that reducing the percentage of sand and increasing the percentage of silt (39.78% on average in the three elevation) in this site relative to Dareshahr, and the semi-heavy texture (loamy) play a key role in the lower amount of total phenol in this site compared to Dareshahr, which contradicts the results of^42^ for the *Gundelia tournefortii* species.

Furthermore, elevation above sea level had a significant effect on the amount of phenol, by the results of^13,38,45,48,54^. In Baderh, with increasing elevation, the amount of phenol increased and reached its highest point at the highest altitude. In many herbaceous species, it has been shown that the synthesis of certain derivatives of the phenolpropanoidide pathway, including flavonoids such as flavanones, flavonols, and also anthocyanins, are encouraged in response to UV^63^. Among the living organisms, plants are more vulnerable to UV due to their inevitable need for photosynthesis^53^. Plants vary in terms of UV sensitivity, and this difference is due to differences in plant species, the variety in agriculture, growth stages, the source of light, exposure time, and environmental conditions. UV targets in plants include proteins, biomembranes, photosynthetic pigments, optical photocysts, plant hormones, and DNA^64^. It has been determined that to protect the internal tissues of leaves and stem from the destructive effects of ultraviolet radiation, absorbent flavonoids, especially flavonols and anthocyanins, accumulate in the surface of epidermal cells. These compounds are potent inhibitors of ROSs (Reactive Oxygen Species) and thus prevent the peroxidation of lipids in plant tissues. It has been reported that the factor of elevation affects the amount of secondary metabolites in plants. Also, it causes many climate differences. Increases in phenolic compounds with elevation might be a response to the increase in UV rays^63,65^). However, in Dehloran, with increasing altitude, the amount of phenol decreased and reached its lowest point in the site. Furthermore, in Dareshahr, elevation did not have any effect on the amount of phenol. Soil and climatic characteristics in these two sites appear to have had more of an impact on the amount of phenol.

In contrast, the interaction between site and elevation had a significant effect on the phenol content of the plant, which is consistent with the findings of^38^. The highest amount of total phenol (55.7689 ± 3.17 μg/mL) was observed for the middle elevation of the Dareshahr site. The acidity, potassium concentration, lime, and sand percentage, which correlate directly with the amount of phenol in this site, reached their highest level at mid-elevation. Also, the electrical conductivity and silt content of the soil at this elevation were significantly lower than at the two other elevations. However, the elevation did not affect the amount of total phenol. Additionally, the lowest total phenol content (25.6644 ± 21.3 μg/mL) was recorded at the lowest elevation of the Badreh site, which had the lowest amount of phosphorus, potassium, organic carbon, organic matter, and nitrogen and the lowest acidity, and lime and silt percentage. Temperature regulates the biosynthetic pathway of phenolic compounds both at high and low temperatures. A reduction in these compounds at high temperatures can be due to a reduction in mRNA transcription^27^. In Dehloran, electrical conductivity had a strong direct correlation with the total phenol content. At the lowest elevation in this site, the highest electrical conductivity (5.26 ± 0.059 ds/m) was recorded, which increased the total phenol content relative to the other elevations of the site. In the study by^14^ on *Dittrichia graveolens* L.^44^, on *Elaeagnus angustifolia* L., and^52^ on *Cichorium intybus* L., salinity also increased the amount of phenol. Plants either avoid salinity or tolerate it. As part of the plant’s resistance to saltiness, the plant absorbs salt and sends it to the organs, especially the leaves. Therefore, due to increased salt stress, flavonoids and phenols increase^66^. A secondary aspect of the salinity problem in plants is the induction of the production of free oxygen radicals, which increases under stress in the plant and affects macro-molecules in the cellular interior causing them to degrade. The plant uses antioxidants to combat these types of radicals^67,68^. Increasing the amount of active oxygen radicals in the plant activates various mechanisms in the plant to reduce the toxic effects of oxidative stress caused by salinity stress. Under these conditions, the amount of antioxidants increases and ROS inhibitors increase to reduce the toxic effects of oxidative stress due to salinity stress^69^. Therefore, it can be seen that in dry and warm climate conditions, unlike moderate semi-arid conditions, soil and especially salinity, is an effective factor in increasing a plant’s phenol content.

### Correlation between antioxidant activity and total phenol content

The results indicate that there is a significant correlation between antioxidant activity and the total phenol content in the two valleys. The results of many studies, such as^62,70–74^ also confirm this correlation. The antioxidant activity of phenols is mainly due to their oxidation properties and their reduction, which allows them to act as reducing agents and suppliers of hydrogen and oxygen. The key role of phenolic compounds has been reported as the removal of free radicals^75,76^. Antioxidants are divided into two main groups based on their performance: primary and secondary antioxidants. Primary antioxidants act to donate an electron or hydrogen to radicals, whereas secondary antioxidants act as an aidant, by providing hydrogen and recovering the primary antioxidants^77^. Phenols and flavonoids are commonly used to remove free radicals^78,79^. Phenolic OH groups are one of the preferred groups for the loss of a proton from single oxidized forms. The stability of the resulting phenoxyl radicals increases their antioxidant properties and the ability of most compounds containing multiple hydroxyl groups to eliminate oxidized free radicals, as well as preventing the formation of free radicals due to lipid peroxidation^80^.

However, there is a strong correlation between the total phenol content and plant antioxidant activity in Dehloran. This phenomenon shows that the presence of other non-phenol compounds, especially flavonoids, contributes to the antioxidant properties of this population. Studies have shown that in addition to phenolic compounds, other factors also affect the level of antioxidant activity. Therefore, due to the complexity of the compounds in the plants, it is difficult to establish a relationship between the antioxidant activity and the particular plant compounds^81^. Polar and non-polar collections seem to be involved in creating antioxidant properties. Of course, further research to identify the components of the extract of this plant will help to detect antioxidant compounds^45^ also achieved similar results in *Dianthus* sp. In some plants, the antioxidant activity might be due to unknown compounds or synergistic interactions between different materials. In addition to saponins, phenols and flavonoids are also known as antioxidants. Each plant has a wide range of phenolic compounds and the antioxidant properties of each of these substances depend on their chemical structure. For example, the antioxidant activity of flavonoids increases with an increase in the number of hydroxyl groups attached to the B ring, especially on carbon, the position of ‘3’, or a single hydroxy substituent^82^.

Therefore, phenolic compounds probably comprise an important part of the antioxidant compounds of this plant, which grows in semi-arid and temperate climates in Dareshahr, at higher altitudes with lower temperatures of the mud. However, in the warm and dry sites of Dehloran, salinity was one of the important factors in the formation of phenol. As the elevation increased, the concentration of soil salts decreased and the temperature increased; the synthesis of phenols decreased and the antioxidant properties of the plant were guided by the formation of other compounds, which should be identified in further investigations of these compounds.

## Conclusion

Location in nature is one of the main factors that can affect the quantity and quality of a plant’s material. The present study showed that ecological factors (climate, elevation, and soil characteristics) have a significant effect on the efficiency of the extract, antioxidant activity, and total phenol content of *S. striata*. Different factors produce different results, and the dominance of different factors in different places caused variation in the results, which indicates the complexity of the effects of ecological factors and the occurrence of various chemical processes in the plant affected by these factors, which causes the synthesis of various compounds that affect the antioxidant properties of the plant. Overall, the Dareshahr site had the highest yield, antioxidant activity, and total phenol content. The best place to grow *S. striata* to produce the highest level of phenolic compounds and antioxidant properties is at the second altitude (600 m above sea level) in Dareshahr.
